# Repurposing of MitoTam: Novel Anti-Cancer Drug Candidate Exhibits Potent Activity against Major Protozoan and Fungal Pathogens

**DOI:** 10.1128/aac.00727-22

**Published:** 2022-07-20

**Authors:** Dominik Arbon, Kateřina Ženíšková, Karolína Šubrtová, Jan Mach, Jan Štursa, Marta Machado, Farnaz Zahedifard, Tereza Leštinová, Carolina Hierro-Yap, Jiri Neuzil, Petr Volf, Markus Ganter, Martin Zoltner, Alena Zíková, Lukáš Werner, Robert Sutak

**Affiliations:** a Department of Parasitology, Faculty of Science, Charles University, BIOCEV, Vestec, Czech Republic; b Institute of Biotechnology, Czech Academy of Sciences, BIOCEV, Vestec, Czech Republic; c Centre for Infectious Diseases, Parasitology, Heidelberg University Hospital, Heidelberg, Germany; d Graduate Program in Areas of Basic and Applied Biology, Instituto de Ciências Biomédicas Abel Salazar, Universidade do Porto, Portugal; e Faculty of Sciences, Charles University, Department of Parasitology, Prague, Czech Republic; f Institute of Parasitology, Biology Centre, Czech Academy of Sciences, České Budějovice, Czech Republic; g School of Pharmacy and Medical Science, Griffith University, Southport, Queensland, Australia; h Department of Physiology, Faculty of Science, Charles University, Prague, Czech Republic; i Faculty of Science, University of South Bohemia, České Budějovice, Czech Republic

**Keywords:** *Candida*, *Cryptococcus*, drug, *Leishmania*, mitochondria, *Plasmodium*, *Trypanosoma*

## Abstract

Many of the currently available anti-parasitic and anti-fungal frontline drugs have severe limitations, including adverse side effects, complex administration, and increasing occurrence of resistance. The discovery and development of new therapeutic agents is a costly and lengthy process. Therefore, repurposing drugs with already established clinical application offers an attractive, fast-track approach for novel treatment options. In this study, we show that the anti-cancer drug candidate MitoTam, a mitochondria-targeted analog of tamoxifen, efficiently eliminates a wide range of evolutionarily distinct pathogens *in vitro*, including pathogenic fungi, Plasmodium falciparum, and several species of trypanosomatid parasites, causative agents of debilitating neglected tropical diseases. MitoTam treatment was also effective *in vivo* and significantly reduced parasitemia of two medically important parasites, Leishmania mexicana and Trypanosoma brucei, in their respective animal infection models. Functional analysis in the bloodstream form of T. brucei showed that MitoTam rapidly altered mitochondrial functions, particularly affecting cellular respiration, lowering ATP levels, and dissipating mitochondrial membrane potential. Our data suggest that the mode of action of MitoTam involves disruption of the inner mitochondrial membrane, leading to rapid organelle depolarization and cell death. Altogether, MitoTam is an excellent candidate drug against several important pathogens, for which there are no efficient therapies and for which drug development is not a priority.

## INTRODUCTION

The ongoing search for novel treatment options to combat medically relevant parasitic protists (e.g., *Plasmodium*, *Trypanosoma,* and *Leishmania* spp.) is driven by the need for more efficient, less toxic, and/or less expensive medications as well as by the emergence and spread of drug resistance (1–3). Drug discovery and development have been facilitated in recent decades by advances in the fields of genetics, molecular biology, medicinal chemistry and the introduction of high-throughput target-based, phenotypic and virtual screening strategies. Nevertheless, repurposing of drugs originally approved for other indications presents a strategy of particular interest for the implementation of novel treatments for neglected diseases (3). As repurposed drugs are typically at least partly characterized or even approved for clinical use, both the time and cost of the drug development process are dramatically reduced. This is particularly appealing for drugs against neglected diseases with little financial incentive for commercial ‘*de novo’* drug discovery approaches.

Phosphonium salts are lipophilic cations with the ability to readily pass across phospholipid bilayers, and due to their delocalized positive charge, they accumulate at the interface of the inner mitochondrial membrane (IMM) and matrix according to the mitochondrial membrane potential (ΔΨ_m_). The extent of accumulation of lipophilic cations at the IMM follows the Nernst equation, according to which there is a 10-fold increase in the concentration of lipophilic cations at the IMM-matrix interface for every ~60 mV increase in ΔΨ_m_ (4). Phosphonium vectors have been employed for efficient and selective mitochondrial delivery of various cargos such as therapeutic antioxidants (5, 6), liposomes (7), functional probes (8, 9), antimicrobial, anti-fungal and anti-parasitic drugs (10–16) and anti-cancer treatments (10, 17, 18).

MitoTam, a new tamoxifen derivative conjugated to triphenylphosphonium vector (TPP+), is a promising anti-cancer drug candidate acting by mitochondrial destabilization (19). It was originally developed to selectively accumulate the pharmacophore tamoxifen in the mitochondria proportionally to ΔΨ_m_. The compound is well tolerated in the mouse model and recently underwent a phase 1/1b clinical trial with favorable outcome when it was intravenously administered to human advanced-stage cancer patients (MitoTam-01 trial; EudraCT 2017-004441-25). Its molecule consists of three parts: i) the functional pharmacophore residue, tamoxifen, a drug that has been used for decades to treat early and advanced hormone-dependent breast cancer (20), ii) the TPP^+^ for mitochondrial targeting, and iii) the 10-carbon linear alkyl linker tethering tamoxifen to the TPP^+^ moiety. The compound efficiently kills breast cancer cells and suppresses tumors progression, including treatment-resistant carcinomas with high Her2 protein levels (Her2^high^), for which the original precursor compound tamoxifen was ineffective. Importantly, MitoTam exhibits low toxicity toward nonmalignant cells (19). Similar to the mitochondria-mediated anti-cancer effects of tamoxifen, MitoTam modulates or alters multiple mitochondrial processes, including the function of the respiratory complex I (CI; NADH:ubiquinone dehydrogenase) (19, 21–23). The superior efficacy of MitoTam compared to tamoxifen is due to its accumulation at the interface of the mitochondrial matrix and IMM that leads to suppression of CI-dependent respiration, disruption of respiratory supercomplexes, rapid dissipation of the ΔΨ_m_, increased production of reactive oxygen species (ROS), and ultimately cell death (19). MitoTam is also effective in the treatment of the syngeneic renal cancer murine model, especially in combination with immunotherapy (23). In addition, MitoTam selectively eliminates senescent cells and is, therefore, a candidate for the treatment of age-related disorders (22, 24).

In this work, we tested the potential activity of MitoTam against a wide range of parasitic protists and pathogenic fungi (Table S1). These models were selected for their medicinal and economic relevance and for their suitability as cellular models for research of mitochondrial function. We chose parasites from the Kinetoplastida group: Trypanosoma cruzi, an intracellular parasite responsible for Chagas disease; *Leishmania* spp., the etiological agent of a spectrum of disorders ranging from self-healing cutaneous lesions to potentially fatal visceral diseases; and Trypanosoma brucei, a highly tractable model organism causing major economical complications in the developing world due to livestock infections and a causative agent of human African trypanosomiasis (sleeping sickness)—a disease that is currently targeted for elimination due to combination of vector control, active case-finding and development of new medicines (25–28).

We also tested the effect of MitoTam on Plasmodium falciparum, a parasite responsible for most malaria-related deaths in humans (29); on the pathogenic fungi Candida albicans and Cryptococcus neoformans, widespread opportunistic pathogens causing life-threatening diseases in immunocompromised individuals (30, 31); on the amphizoic amebae Naegleria fowleri and *Acanthamoeba* spp., whose infections lead to rare diseases with extremely high mortality rate (32, 33); and on Giardia intestinalis and Trichomonas vaginalis, anaerobic parasites with reduced mitochondria, causative agents for widespread intestinal and urogenital infections (34–36). Here, we show that the anti-cancer drug candidate MitoTam efficiently and selectively inhibits viability of a number of these pathogens *in vitro*. In pilot experiments using mouse models of infection with T. brucei and L. mexicana, MitoTam reduced parasite burdens and significantly prolonged survival for T. brucei-infected animals. Furthermore, we demonstrate in T. brucei that the trypanocidal activity of MitoTam relies, at least partly, on rapid disruption of IMM. Together, our data provide strong evidence that MitoTam is an excellent candidate for further development as an anti-infective, especially for targeting several neglected diseases.

## RESULTS

### Low levels of MitoTam are lethal to a variety of pathogenic eukaryotic microorganisms.

The mitochondrion is a promising drug target because of its pivotal role in cellular metabolism, proliferation, and cell death signaling (26, 37, 38). We therefore investigated the effect of MitoTam, a mitochondria-targeted tamoxifen derivative, on a wide range of parasitic protists and yeasts. MitoTam proved to have a significant growth inhibitory effect for the majority of these pathogens (Table 1 and Fig. S1) exhibiting nanomolar potencies against P. falciparum, *T. b. brucei* and *Leishmania* spp. Notably, the half-maximal effective concentration (EC_50_) values are considerably lower than corresponding cytotoxicity values reported for various mammalian cells (ranging from 0.6 to 4.5 μM) (19), indicating high selectivity. All pathogens were also challenged with the parental compound tamoxifen lacking the TPP^+^ vector for mitochondrial targeting, with efficacy significantly lower than MitoTam (Table 1). This is consistent with similar comparisons reported for the anti-cancer activity of the two molecules (19). Each dose-response analysis was validated using a known, active compound against individual pathogens tested, providing values consistent with published data (Table 1).

**TABLE 1:** MitoTam inhibits the growth of several pathogenic microorganisms *in vitro*^*a*^

Pathogen	MitoTam		Tamoxifen		Control		
	EC_50_ [μM]	SD	EC_50_ [μM]	SD	Compound	EC_50_ [μM]	SD
Trypanosoma brucei BSF	0.02	≤0.01	7.20	±1.47	Amphotericin B	1.09	±0.09
Trypanosoma brucei PCF	0.16	±0.03	≥10		Amphotericin B	0.24	±0.06
*T. brucei gambiense* BSF	0.03	≤0.01	≥10		Pentamidine	≤0.01	≤0.001
Trypanosoma cruzi (epimastigotes)	1.55	±0.04	≥10		Benznidazole	≥10	
Leishmania mexicana (amastigotes)	0.35	±0.05	7.93	±0.59	Amphotericin B	0.33	±0.08
Leishmania major (promastigotes)	0.60	±0.10	≥10		Amphotericin B	0.06	±0.02
Plasmodium falciparum (erythrocytic stage)	0.03	±0.01	6.37	±0.11	Chloroquine	0.01	≤0.01
Candida albicans	0.56	±0.07	≥10		Amphotericin B	0.37	±0.01
Cryptococcus neoformans	0.61	±0.12	7.28	±1.97	Amphotericin B	0.12	±0.05
Naegleria fowleri	≥10		≥10		Amphotericin B	0.08	±0.01
Acanthamoeba castellanii	1.95	±0.23	9.93	±0.58	Amphotericin B	6.17	±0.70
Giardia intestinalis	≥10		≥10		Benznidazole	6.22	±0.66
Trichomonas vaginalis	≥10		≥10		Metronidazole	6.54	±0.45

a Mean EC_50_ values for MitoTam, tamoxifen (the functional pharmacophore of MitoTam) and specific antimicrobial drugs used as positive controls for each tested organism derived from dose-response curves (Fig. S1) are given in μM (n > 3, ± s.d.).

The highest efficacy of MitoTam was detected for the bloodstream form (BSF) of T. brucei. This value was approximately 30-fold lower than Her2^high^ cancer cells (0.02 μM *versus* 0.65 μM) (19). Interestingly, MitoTam showed low nanomolar efficacy (0.03 μM) against the erythrocytic stage of P. falciparum, which is protected inside the parasitophorous vacuole within the host erythrocyte (39), suggesting that MitoTam is able to cross several membranes before reaching its target. Furthermore, MitoTam was found to inhibit the growth of the T. brucei procyclic form (PCF), axenically grown Leishmania mexicana amastigotes and L. major promastigotes, and the two pathogenic fungi Candida albicans and Cryptococcus neoformans at submicromolar EC_50_ values. T. cruzi epimastigotes also responded to MitoTam treatment with low-micromolar EC_50_, as did *Acanthamoeba* sp., while no effect was observed on Naegleria fowleri proliferation. Also, Trichomonas vaginalis and Giardia intestinalis, two anaerobic parasites that possess mitochondrion-related organelles characterized by the absence of membrane-bound electron transport chain and therefore by the lack of mitochondrial respiration (36), were insensitive to MitoTam treatment. Overall, MitoTam effectively killed a range of clinically important, evolutionarily distinct pathogens, including intracellular parasites.

### MitoTam efficiently eliminates L. major and L. infantum intracellular amastigotes.

The high efficacy of MitoTam against L. mexicana axenic amastigotes (EC_50_ 0.35 ± 0.05 μM) prompted us to investigate the ability of MitoTam to eliminate the intracellular form of the parasite in a macrophage infection model. Murine macrophage cells (J774A.1) were infected with L. major or L. infantum promastigotes and exposed to different concentrations of MitoTam (0 to 25μM). After controlled lysis of the infected macrophages, the released parasites were quantified using a resazurin-based viability assay (40). In this intramacrophage assay, treatment with MitoTam eliminated intracellular amastigotes of L. major and L. infantum with EC_50_ values of 175 ± 51 nM and 293 ± 29 nM, respectively (Fig. S2). This result is highly encouraging considering how difficult is to effectively target the intracellular *Leishmania* amastigotes *in vivo* since the parasites reside in a parasitophorous vacuole, a phagolysosome-like structure with low pH and a potential drug target is protected by three membranes (plasma membrane of a macrophage, parasitophorous vacuolar membrane and plasma membrane of an amastigote) (26).

### MitoTam treatment alleviates parasitemia leading to prolonged survival of mice infected with T. brucei and to reduced frequency and lesion size caused by L. mexicana infection.

Activity for a compound detected in the intramacrophage assay usually translates into activity in an animal infection model (41, 42), apart from potential issues with pharmacokinetics. Hence, we next tested the ability of MitoTam, which is well tolerated in BALB/c mice (19), a mouse infection model of both L. mexicana and T. brucei. MitoTam dosing regimen was based on the published data (19, 22).

**FIG 1:**
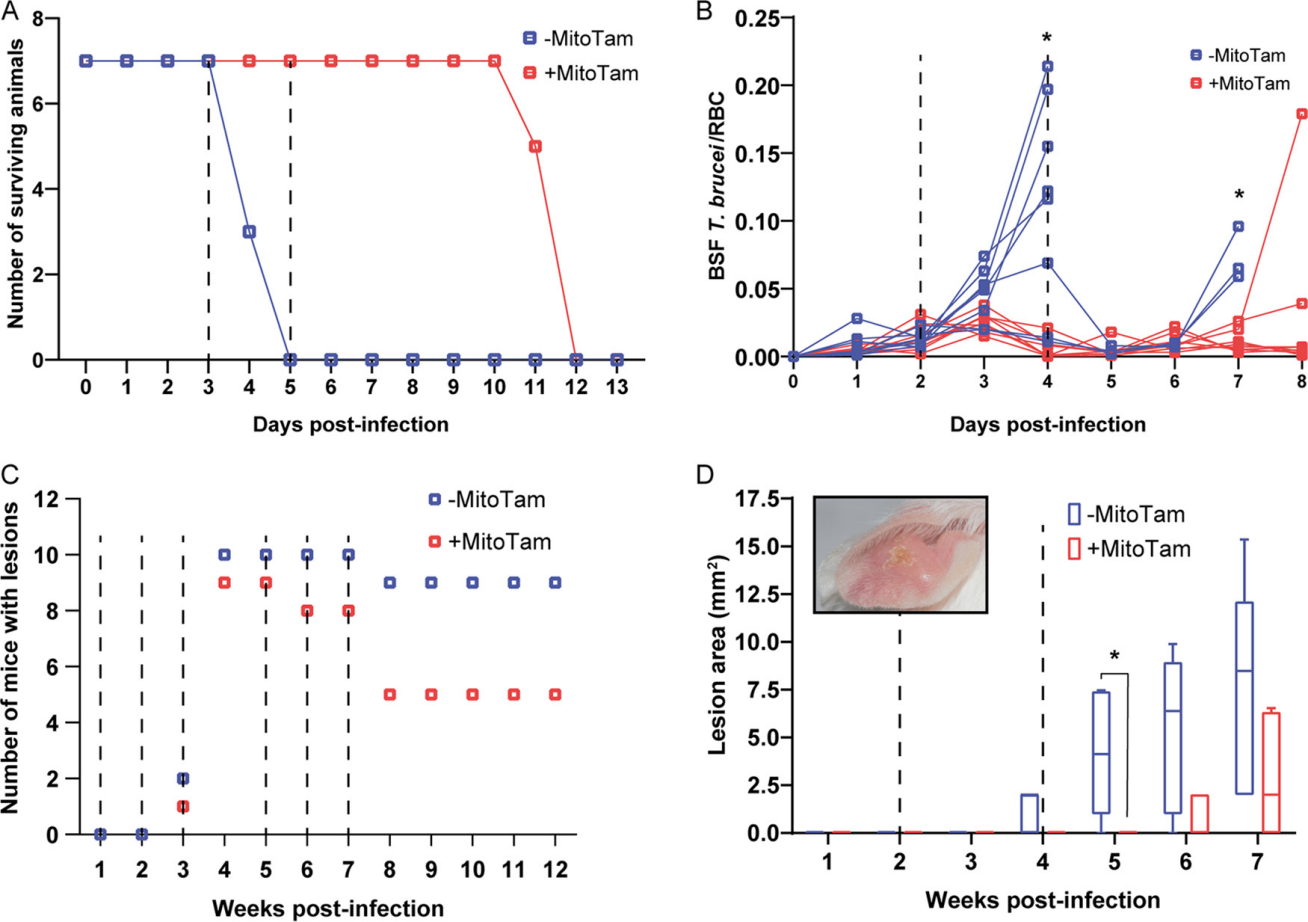
MitoTam is effective against T. brucei and L. mexicana parasites in mouse infection models. (A) Survival rate of BALB/c mice infected with T. brucei was monitored daily for 13 days. Half of the infected mice were injected IV with MitoTam (+MitoTam) on days 3 and 5 (dashed lines) postinfection (3 mg/kg bw). The number of surviving mice from MitoTam treated group (red line) as well as from untreated control group (-MitoTam) (blue line) is plotted against the time (in days). (B) Parasitemia in BALB/c mice infected with T. brucei was evaluated microscopically from blood smears. On day 2 and 4 postinfection (dashed lines), eight mice were IV injected with MitoTam (3 mg/kg) (+MitoTam). The ratio of T. brucei cells to red blood cells (RBC) was calculated daily and used to quantify the parasitemia levels of each animal from the MitoTam treated group (red line) and the respective untreated control group (-MitoTam) (blue line). Statistically significant differences between the two groups are indicated with an asterisk (*unpaired t test*, *, *P* < 0.05). (C) Formation of dermal lesions in 20 BALB/c mice was followed for 12 weeks after infection with L. mexicana. As indicated (dashed lines), half of the infected animals were injected IP with MitoTam (+MitoTam) at 1, 2, 3, 5, 6 and 7 weeks postinfection. The appearance of lesions was monitored (+MitoTam, red) and compared to the number of lesions of animals from the untreated control group (-MitoTam, blue). (D) Size of dermal lesions was recorded in 10 BALB/c mice infected with L. mexicana. Five of the infected animals were treated with MitoTam (+MitoTam) at weeks 2 and 4 postinfection as indicated (dashed lines). Throughout the course of the infection, the lesion areas of each animal were measured weekly. The values were averaged for the MitoTam treated group (+MitoTam, in red) and untreated control group (-MitoTam, in blue). Box and whisker graph is shown, statistically significant differences are indicated with an asterisk (paired *t* test, *, *P* < 0.05). A representative lesion image is shown as an inset in the graph.

For the T. brucei infection model, survival analysis revealed that MitoTam intravenous (IV) administration at doses of 3 mg/kg body weight (bw) on days 3 and 5 postinfection without further treatment delayed the death of T. brucei infected animals, which succumb to the infection by 8 days (Fig. 1A).

In a second experiment, we followed T. brucei parasitemia levels in infected animals (Fig. 1B). Automated analysis of blood smears revealed the presence of T. brucei parasites in samples from mice untreated and treated on days 2 and 4 postinfection, showing that MitoTam-treated animals exhibited a significantly lower parasite load (Fig. 1B). This explains the longer survival of treated animals compared to the untreated controls (Fig. S3).

The effect of MitoTam administration (3 mg/kg bw, intraperitoneally [IP]) in the L. mexicana infection model was assessed by monitoring frequency and size of dermal lesions. As shown in Fig. 1C, lesions occurred less frequently in MitoTam-treated BALB/c mice than in the untreated controls during the 12-week course of the experiment (50% versus 90% of mice at weeks 8 to 12 postinfection). In a separate experiment, MitoTam at two doses reduced the size of lesions caused by L. mexicana in the weeks following infection (Fig. 1D).

Collectively, these data demonstrate that MitoTam is highly effective against T. brucei and L. mexicana parasites *in vivo*. Notably, we did not observe any notable adverse effects of MitoTam on the treated animals.

### MitoTam treatment causes alteration of T. brucei mitochondrial proteome and leads to upregulation of multidrug efflux pumps in C. albicans.

**FIG 2:**
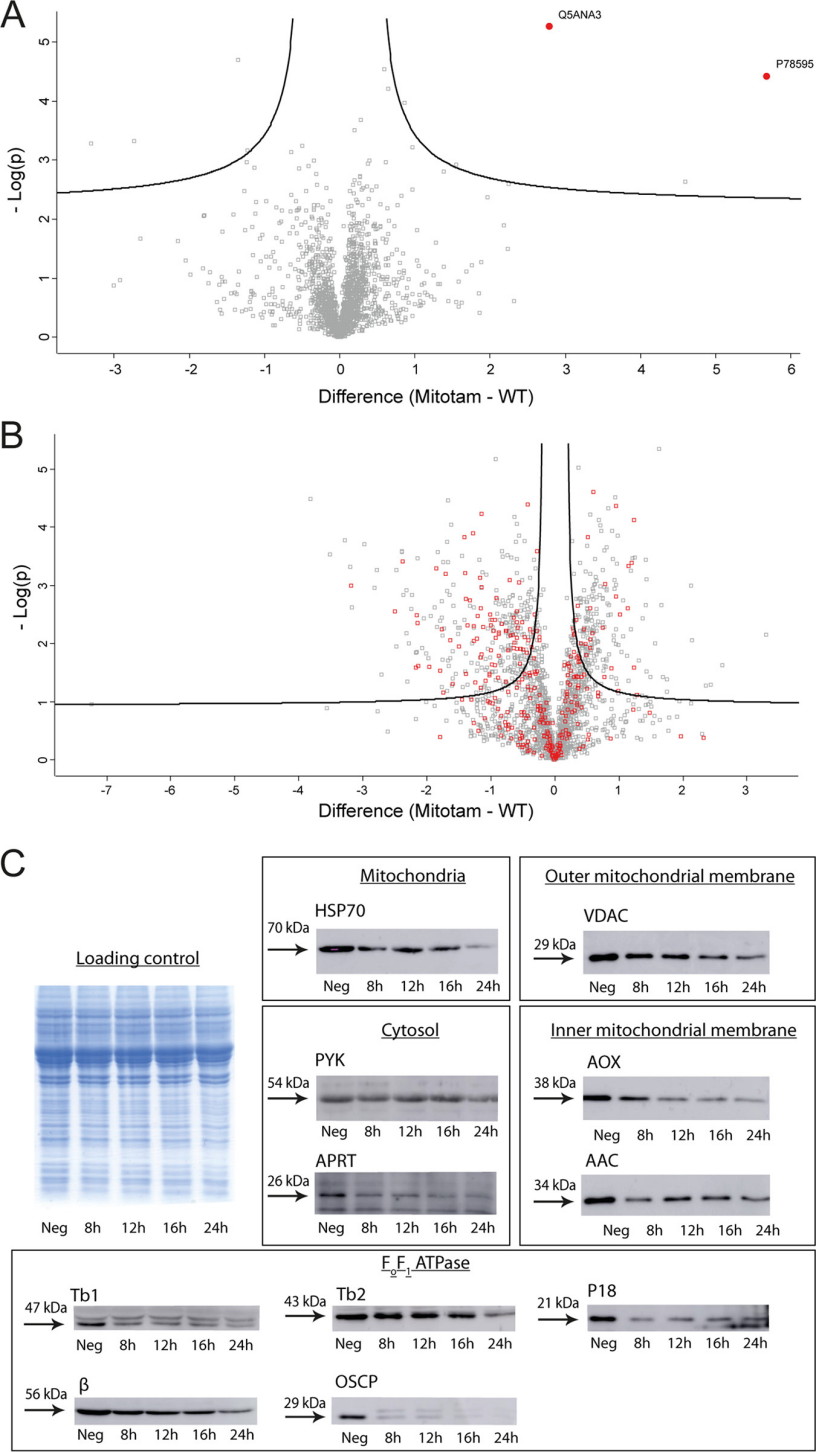
Global proteome changes upon MitoTam exposure. Shown are volcano plots of the *t* test difference from label-free quantification from triplicate experiments, plotted against the respective −log_10_-transformed *P* values. Untreated control cells were compared to cells treated with MitoTam for C. albicans (A) or BSF T. brucei (B). (A) Highlighted points are C. albicans significantly upregulated efflux pumps (red dots): P78595—multidrug resistance protein CDR2; Q5ANA3—pleiotropic ABC efflux transporter of multiple drugs CDR1. (B) T. brucei mitochondrial proteins are in red. (C) Western blot analyses of whole-cell lysates harvested from BSF T. brucei cells treated with 100 nM MitoTam for 0 to 24 h as indicated. Equal amounts of total protein were loaded in each lane. A Coomassie stained gel was used as a control for equal loading. The immunoblots were probed with antibodies against cytosolic pyruvate kinase (PYK) and adenine phosphoribosyltransferase (APRT) and mitochondrial heat shock protein 70 (Hsp70), proteins associated with the outer mitochondrial membrane (VDAC), inner mitochondrial membrane (AOX, AAC), and subunits of the F_o_F_1_-ATP synthase (β, p18, OSCP, Tb1, Tb2). The relevant masses of the protein molecular weight are indicated.

The potent anti-parasitic properties *in vitro* and the promising *in vivo* experiments prompted us to explore the MitoTam mode of action. First, we performed comparative whole-cell proteomic analysis of control and MitoTam-treated cells for two parasitic model organisms, C. albicans and T. brucei. In order to restrict indirect, secondary impact resulting from cell death, we chose short exposure times: yeast cells were treated with 4.4 μM MitoTam (~7× EC_50_) for 12 h, while T. brucei BSF cells were incubated with 100 nM MitoTam (5× EC_50_) for 14 h. Proteomic data were processed by label-free quantification in MaxQuant (43) and statistically analyzed in Perseus (44, 45). In C. albicans, out of 1,950 detected protein groups, the abundance of only 1.69% of the quantified proteins was significantly altered (Table S2) (Fig. 2A), with two homologs of multidrug efflux pumps being among the most upregulated genes (≥26 times).

In contrast, analysis of the T. brucei proteome revealed substantial changes after treatment with MitoTam, with 26.3% of all identified proteins (2,063) significantly altered (Table S3) (Fig. 2B). Data analyses did not reveal any specific single pathway or protein as a direct target of MitoTam. Since MitoTam specifically affects mitochondrial functions in various cells (19, 22, 23), we analyzed enrichment of mitochondrial proteins using a T. brucei mitoproteome database (46). While mitochondrial proteins accounted for 14.3% of all detected proteins, we observed an almost 2-fold enrichment (27.6%) in the significantly altered subset of proteins, with 20.8% and 32.1% in the increased and decreased cohort, respectively. These results indicate that MitoTam treatment induces preferentially a decrease in the levels of various mitochondrial proteins. To validate these results, we analyzed T. brucei whole-cell lysates from cultures harvested at 4 different time points of MitoTam exposure (8, 12, 16, and 24 h) by immunoblotting with antibodies against 8 mitochondrial marker proteins and cytosolic pyruvate kinase as a control (Fig. 2C). Consistent with the T. brucei proteomics analysis, we detected significant abundance decrease for nine mitochondrial proteins after 8 h of MitoTam treatment.

### MitoTam treatment leads to disruption of the mitochondrial inner membrane and rapid loss of ΔΨ_m_ in the bloodstream form of T. brucei.

MitoTam was reported to directly impact mitochondrial function in renal and breast cancer cells, tumors, and senescent cells (19, 22, 23). In line with this, our proteomic analysis indicated that MitoTam treatment perturbs the mitochondrial proteome in T. brucei. T. brucei BSF cells lack cytochrome-mediated mitochondrial electron transport chain, respiring exclusively via an alternative pathway consisting of mitochondrial glycerol-3-phosphate dehydrogenase and trypanosome alternative oxidase (AOX), and sustain the ΔΨ_m_ by reversed activity of F_o_F_1_-ATP synthase (47–49). Importantly, the cancer molecular target of MitoTam, respiratory CI (19) was shown to be dispensable for BSF cells (50), yet our data show that the drug efficiently eliminates these parasites.

**FIG 3:**
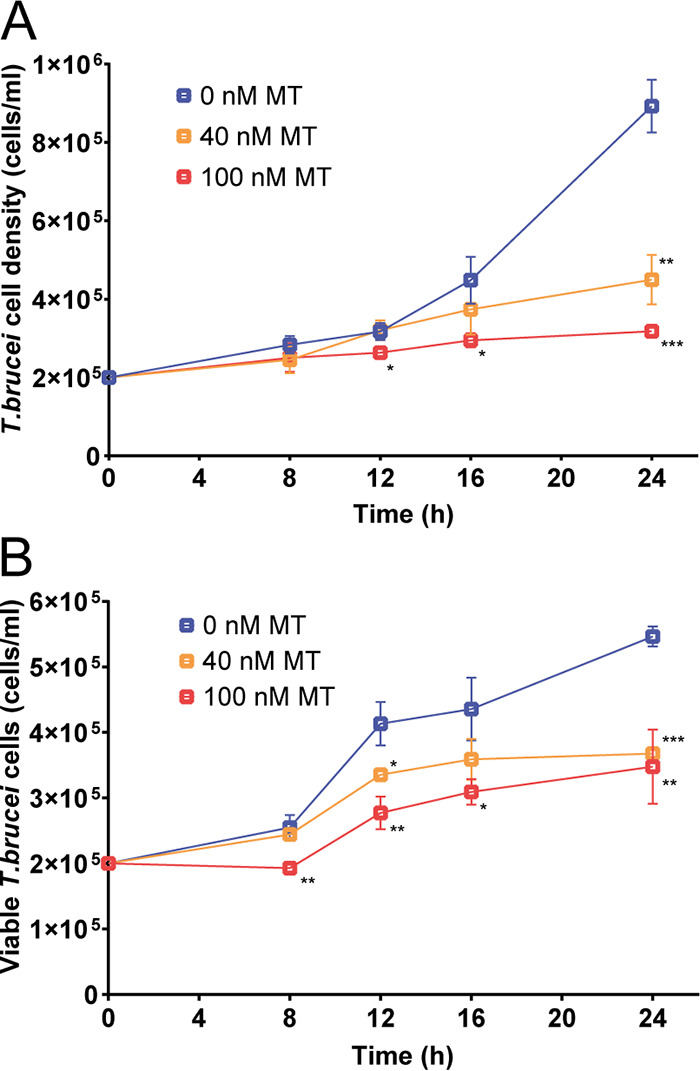
MitoTam affects T. brucei viability and alters the mitochondrial function of bloodstream T. brucei cells. (A) Growth curve of BSF T. brucei cultures was evaluated in unstained cells by flow cytometry. Untreated control BSF cells (0 nM MT, blue) were compared with cells incubated with 40 nM MitoTam (40 nM MT, orange) and 100 nM MitoTam (100 nM MT, red). Statistically significant differences are indicated with an asterisk (paired *t* test, *, *P* < 0.05, **, *P* < 0.01, ***, *P* < 0.001) (mean ± s.d., *n* = 3). (B) Staining with the cell impermeant nucleic acid dye Sytox was used to distinguish viable (Sytox negative) and dead (Sytox positive) BSF T. brucei cells. Concentrations of Sytox negative, live cells are plotted. Untreated control cultures (0 nM MT, blue) were compared with cultures incubated in the presence of 40 nM MitoTam (40 nM MT, orange) and 100 nM MitoTam (100 nM MT, red) (paired *t* test, *, *P* < 0.05, **, *P* < 0.01, ***, *P* < 0.001) (mean ± s.d., *n* = 3).

**FIG 4:**
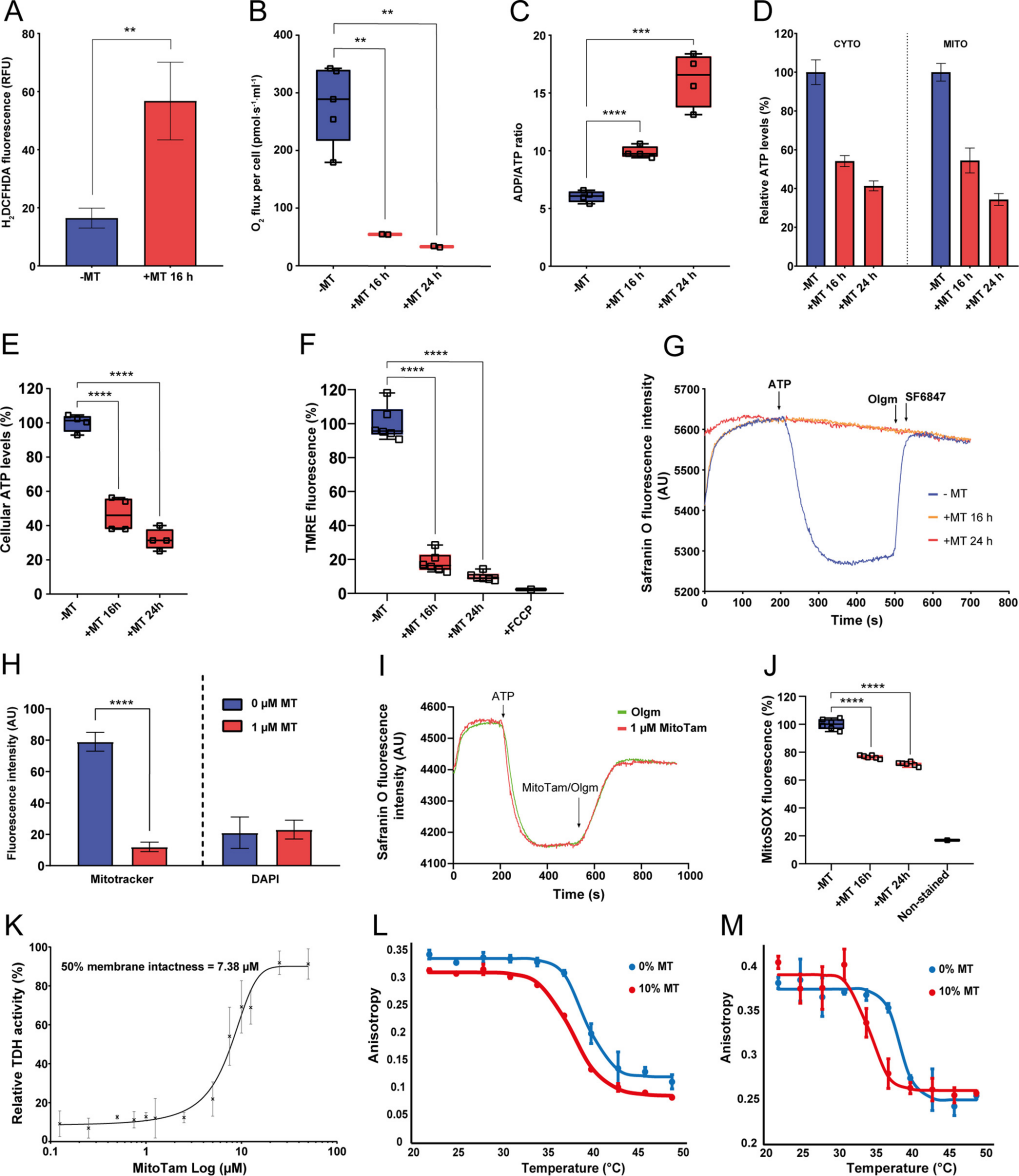
Overall effect of MitoTam on the T. brucei cell. (A) Production of intracellular ROS in BSF T. brucei was quantified by flow cytometry using DCFH-DA detection reagent in untreated control cells (−MT, blue) and cells treated with 100 nM MitoTam for 16 h (+MT 16 h, red) (mean ± s.d., *n *= 3, **, *P* < 0.01). (B) The O_2_ flux per cell using high-resolution respirometry after addition of glycerol-3-phosphate was determined in BSF T. brucei untreated control cells (−MT, blue) and BSF cells treated with 100 nM MitoTam for 16 h (+MT 16 h, red) and 24 h (+MT 24 h, red) (box and whisker plot, *n *= 3, **, *P* < 0.01). (C) Relative ADP/ATP ratio analyzed using a bioluminescence assay kit in BSF T. brucei untreated control cells (−MT, blue) and BSF cells treated with 100 nM MitoTam for 16 h (+MT 16 h, red) and 24 h (+MT 24 h, red) (box and whisker plot, *n *= 3, ***, *P* < 0.001, ****, *P* < 0.0001). (D) Cytosolic and mitochondrial ATP levels were assessed in transgenic BSF T. brucei cell lines expressing firefly luciferase. Untreated control cells (−MT, blue) were compared with cells treated with 100 nM MitoTam for 16 h (+MT 16 h, red) and 24 h (+MT 24 h, red). Data were normalized to the values of the untreated control cells and expressed as a percentage (mean ± s.d., *n *= 4). (E) Total cellular ATP levels in BSF T. brucei were determined using a bioluminescence assay kit. Data from cultures treated with MitoTam for 16 h (+MT 16 h, red) and 24 h (+MT 24 h, red) were normalized to the respective values of the untreated control cells (−MT, blue) and expressed in percentage (box and whisker plot, *n* = 4), ****, *P* < 0.0001). (F) Flow cytometry of TMRE stained cells was used to determine ΔΨ_m_ of BSF T. brucei. Data from cultures treated with 100 nM MitoTam for 16 h (16 h +MT, red) and 24 h (+MT 24 h, red) were normalized to the respective values of the untreated control cells (−MT, blue) and expressed in percentage. Uncoupler FCCP was added as a control for ΔΨ_m_ depolarization (box and whisker plot, *n* = 6, ****, *P* < 0.0001). (G) *In situ* ΔΨ_m_ was measured in digitonin-permeabilized BSF T. brucei cells stained with Safranin O dye. Where indicated, the F_o_F_1_-ATP synthase substrate—ATP, the F_o_F_1_-ATP synthase inhibitor—oligomycin (Olgm) and the protonophore SF6847 were added. Representative trace from the measurement of untreated control cells (−MT, blue) in comparison with cells treated with 100 nM MitoTam for 16 h (+MT 16 h, orange) and 24 h (+MT 24 h, red) is shown. (H) Automated microscopic analysis of BSF T. brucei cells stained with Mitotracker Red CMX-ROS was used to determine ΔΨ_m_ in control untreated cells (0 μM MT) and in cells treated with 1 μM MitoTam for 1 h (1 μM MT). Staining with DAPI was used as an internal control for the analysis. Mean signal intensities of Mitotracker Red CMX-ROS and DAPI are depicted on the *y* axis (mean ± s.d., ****, *P* < 0.0001). (I) *In situ* ΔΨ_m_ was measured in digitonin-permeabilized BSF T. brucei cells stained with Safranin O dye. Where indicated, the F_o_F_1_-ATP synthase substrate—ATP, the F_o_F_1_-ATP synthase inhibitor—oligomycin (Olgm, green line) and MitoTam (1 μM, red line) were added. (J) Red mitochondrial superoxide indicator MitoSOX was used to detect intramitochondrial ROS production in untreated BSF T. brucei control cells (−MT, blue) and BSF cells treated with 100 nM MitoTam for 16 h (+MT 16 h, red) and 24 h (+MT 24 h, red). Data were normalized to the values of the untreated control cells and expressed as a percentage (mean ± s.d., *n *= 6, ****, *P* < 0.0001). (K) Integrity of the inner BSF T. brucei mitochondrial membrane was assessed in isolated mitochondria incubated with increasing concentration of MitoTam. The mitochondrial enzyme threonine dehydrogenase (TDH) was used as a marker for permeability of the membrane, as the TDH activity is detectable only if a substrate passes freely through compromised mitochondrial membrane (mean ± s.d., *n *= 3). (L) Phase transition profile of artificial membranes prepared via hydration of lipide film (DPPC + MitoTam [MT] 0 and 10 mol % + 0.1 mol % DPH) with physiological saline and measured over range of temperatures (22 to 49°C). Fluidity change is plotted as a fluorescence anisotropy (r) against temperature. (M) Phase transition profile of artificial membranes prepared via hydration of lipide film (DPPC + MitoTam [MT] 0 and 10 mol % + 0.2 mol % TMA-DPH) with physiological saline and measured over range of temperatures (22 to 49°C). Fluidity change is plotted as a fluorescence anisotropy (r) against temperature.

To gain deeper insight into the anti-parasitic mode of action of MitoTam, we investigated its effect on mitochondrion in this model protist. When comparing the growth of untreated cells with cells treated with two different concentrations of MitoTam, we found that growth of T. brucei cells was significantly inhibited after 24 h (40 nM, 2× EC_50_) or 12 h (100 nM, 5× EC_50_) (Fig. 3A). To establish a timeline for further experiments, we performed a live/dead cell assay using the cell-impermeant dye Sytox. This result shows that despite the reduced growth rate, 82.4% and 71% cells were still viable upon 40 nM and 100 nM MitoTam treatment, respectively, after exposure to the drug for 16 h. At 24 h of treatment, the viability was further decreased to 67.3% and 63.6% at 40 and 100 nM MitoTam, respectively (Fig. 3B). This was accompanied by increased level of cellular ROS of cells treated with 100 nM MitoTam for 16 h (Fig. 4A).

To examine whether MitoTam affects mitochondrial activity, we analyzed changes in several cellular attributes associated with mitochondrial function. T. brucei BSF cultures were treated for 16 and/or 24 h with 40 nM or 100 nM MitoTam. We found that incubation with 100 nM MitoTam significantly reduced glycerol-3-phosphate-dependent respiration, increased cellular ADP/ATP ratio (by 2.6-fold), and decreased mitochondrial and cytosolic ATP as well as total cellular ATP levels (Fig. 4B to E). Next, we assessed ΔΨ_m_ in live cells using the cell-permeable red fluorescent dye TMRE and using the Safranin O assay in digitonin-permeabilized cells. The results of both assays demonstrate that treatment with 100 nM MitoTam rendered the cell incapable of maintaining ΔΨ_m_ (Fig. 4F and G). Experiments with cultures treated with MitoTam at 40 nM showed a similar but less pronounced overall effect on T. brucei mitochondrial activity (Fig. S4).

In agreement with these results, fluorescence microscopy evaluation revealed that treatment with 1 μM MitoTam for 1 h led to an ~8-fold decrease of the signal intensity of MitoTracker Red CMX-ROS, a mitochondrial probe used to stain live cells that relies on ΔΨ_m_ (Fig. 4H).

Furthermore, ΔΨ_m_ was assessed in digitonin-permeabilized wild-type BSF cells by Safranin O dye upon addition of ATP. We found that ΔΨ_m_ was quickly dissipated with 1 μM MitoTam, a phenotype identical to the effect of addition of oligomycin, an inhibitor of ATP synthase (Fig. 4I). Moreover, using mitochondrial superoxide indicator (MitoSOX), we detected a significant decrease of mitochondrial ROS levels upon MitoTam treatment, suggesting that the mode of action of MitoTam in trypanosomes differs from its effect in cancer cells (Fig. 4J).

While the observed changes in the evaluated attributes are consistent with inhibition of F_o_F_1_-ATP synthase that generates ΔΨ_m_ in BSF T. brucei cells (48), we also tested if the rapid drop in ΔΨ_m_ is caused by a physicochemical disruption of the IMM. To this end, we assessed the integrity of isolated mitochondria using the mitochondrial marker threonine dehydrogenase (TDH), whose activity is detectable only if the added substrate threonine can freely pass through ‘compromised’ mitochondrial membrane. As shown in Fig. 4K, treatment with MitoTam at 7.4 μM decreased mitochondrial membrane integrity by 50%. This relatively high value (369 times higher than EC_50_) determined in this *in vitro* experiment can be explained by a need to form much larger membrane pores for threonine (molecular weight 119.12 g/mol) to enter mitochondria compared to the formation of much smaller or temporal pores allowing the passage of H^+^. In addition, in this experiment, the BSF form mitochondrion is not energized due to the lack of substrates and therefore this could have prevented accumulation of MitoTam from in the organelle.

Furthermore, to corroborate these results we estimated the effect of MitoTam to membrane phase transition temperature. The methodology relies on an estimation of degree of free rotation of membrane fluorescent probes *N*,*N*,*N*-trimethyl-4-(6-phenyl-1,3,5,-hexatrien-1-yl), trimenthylammonium *p*-toluenesulfonate (TMA-DPH) and 1,6-diphenyl-1,3,5-hexatriene (DPH) (51, 52). The rigid membrane prevents free rotation of the fluorophore, therefore emitted fluorescence can be vertically and horizontally polarized. Readout of fluorescence anisotrophy (r) then reflects changes in fluidity of membrane subdomains which allows for construction of a thermotropic profile.

The effect of MitoTam (10 mol%) to artificial membrane prepared from dipalmitoylphosphatidylcholine (DPPC) was measured for both the upper bilayer region (0.2 mol% TMA-DPH) as well as for the hydrophobic region (0.1 mol% DPH) across a range of temperatures (22 – 49°C). Indeed, our analysis of the interaction of 10 mol% MitoTam with DPPC liposomes indicated a significant increase in membrane fluidity (Fig. 4L and M). Interestingly, a bigger phase transition temperature shift occurred at the upper membrane region compared to the hydrophobic region (Δ*t* = 1.6°C versus 4.2°C). Although fluorescence polarization measurements require suprapharmacological concentration of the drug these results suggest that modulation of membrane properties likely occurs at interface IMM/matrix or IMM/intermembrane space, whereas the hydrophobic region is less likely to be affected.

Taken together, our data indicate that MitoTam likely changes physicochemical characteristics and disrupts the integrity of the IMM, which leads to the ΔΨ_m_ collapse and cell death.

## DISCUSSION

In cancer cells, MitoTam was shown to inhibit CI-dependent respiration, which leads to a rapid decrease of ΔΨ_m_, increased ROS production, and disruption of respiratory supercomplexes. These effects ultimately trigger cell death. The phenotype is even more pronounced in cancer cells, which exhibit high levels of the Her2 protein in mitochondria and elevated CI-dependent respiration. Consistently, mammalian cells deficient in CI are more resistant to MitoTam (19). In metabolically active senescent cells, MitoTam treatment led to increased levels of ROS, reduced ΔΨm, impaired mitochondrial morphology, and metabolic switching to glycolysis for cellular ATP generation (22). Interestingly, the mitochondrial phenotype was alleviated and cell survival prolonged in MitoTam-treated senescent cells overexpressing adenine nucleotide translocase-2 (ANT2), which imports ATP into mitochondria (22). Furthermore, restriction of glycolytic pathways, either by limiting substrate levels or by adding glycolytic inhibitors competing with glucose sensitized control cells to MitoTam (22). In contrast to cancer cells, ROS scavengers failed to rescue MitoTam-treated senescent cells from death. Moreover, treatment with established CI inhibitors (rotenone and piericidin A) alone did not selectively eliminate senescent cells, while they are sensitive to MitoTam treatment (22). Therefore, the senolytic effect of MitoTam appears to involve additional mechanisms to CI inhibition. Taken together, the effect of MitoTam on cancer and senescent cells seems to be complex and includes targeting of CI, mitochondrial membrane depolarization, loss of ATP, increased levels of ROS, and possibly interplay between ATP/ADP exchange mechanism and ATP synthase (19, 22).

In this study, we showed that MitoTam inhibits the growth of a variety of parasitic protists and fungi. Different sensitivity to the compound may reflect different metabolic adaptations that the pathogens have developed to thrive in their host organism. High potencies were determined for T. brucei BSF and for the erythrocytic stage of P. falciparum, the relevant life cycle stages persisting in the mammalian host of both these medically important parasites. Notably, canonical CI is missing in P. falciparum (53) and is dispensable in BSF T. brucei (50, 54) suggesting that MitoTam has additional molecular targets besides CI. The EC_50_ value of MitoTam was one or 2 orders of magnitude higher (but still in in the micromolar range) for other tested organisms such as *Leishmania*, *Acanthamoeba* sp. and the widespread pathogenic yeasts C. albicans and C. neoformans. MitoTam was the least active against anaerobic protists Trichomonas vaginalis and Giardia intestinalis, likely due to the absence of conventional energized mitochondria in these organisms (36). The low activity observed against facultatively parasitic amebae Naegleria fowleri requires further research.

Following the encouraging results from *in vitro* testing, we demonstrated that MitoTam significantly decreases parasitemia levels, as well as frequency and size of lesions in animals infected with T. brucei and L. mexicana, respectively. In agreement with published data (19, 22, 23), repeated injection of MitoTam had no apparent adverse effect on BALB/c mice used in our studies.

To investigate the mode of action of MitoTam in unicellular pathogens, we performed comparative proteomic analysis using two distinct model organisms, T. brucei (BSF) and C. albicans. Only minor changes were detected in the C. albicans proteome upon MitoTam treatment, the most upregulated proteins being two homologues of multidrug efflux pumps, possibly explaining the 28 times higher EC_50_ value compared to BSF T. brucei cells. The corresponding analysis in T. brucei cells revealed substantial proteome changes upon 12 h MitoTam treatment. While the complexity of the observed alterations failed to pinpoint the exact molecular target(s) of MitoTam, there was a significant impact on the mitochondrion.

To get insight into the complex mode of action that is linked to the function of CI in cancer cells (19), we decided to examine the effect of MitoTam on mitochondrial parameters in BSF T. brucei, where the respiratory complex is dispensable (50). Consistent with studies on cancer and senescent cells (19, 22, 23), we found that cells incubated with MitoTam exhibit increased levels of cellular ROS and resulted in rapid dissipation of ΔΨ_m_, which leads to cell death. In contrast, mitochondrial superoxide levels in T. brucei cells were decreased upon treatment with MitoTam, indicating that the oxidative burst caused by inhibition of CI-dependent respiration is not responsible for T. brucei growth inhibition. Furthermore, T. brucei BSF cells incubated with MitoTam exhibited reduced glycerol-3-phosphate-stimulated oxygen consumption, decreased ATP levels, and increased ADP/ATP ratio, suggesting that the biochemical effect of MitoTam could either be due to inhibition of F_o_F_1_-ATP synthase that generates ΔΨ_m_ in these cells (47, 48) or, simply, due to disruption of the mitochondrial membrane integrity, resulting in ΔΨ_m_ collapse.

Our analysis shows that MitoTam at micromolar concentrations significantly increases the membrane permeability of isolated mitochondria, which could be the cause of the altered mitochondrial phenotype. This effect could be ascribed to a direct interaction of the lipophilic MitoTam molecule with the IMM and alteration in membrane fluidity as demonstrated on artificial liposomal DPPC membrane at suprapharmacological concentration. Disruption or perhaps modulation of the membrane physicochemical properties might indeed rapidly dissipate ΔΨ_m_ in live cells whether it be via unregulated proton leak or via affecting fundamental interactions of IMM and integral proteins, such as F_o_F_1_-ATP synthase.

Alternatively, the effect of MitoTam on membranes may be due to a disruption of enzymes involved in phospholipid synthesis. Intriguingly, studies on T. cruzi, *Leishmania* spp. and P. falciparum indicate that tamoxifen, the functional pharmacophore of MitoTam, has an inhibitory effect on phospholipid metabolic pathways (55–57). Additional experiments are needed to reveal the multifactorial MitoTam mode of action. Nevertheless, our study shows that MitoTam represents a promising candidate to be repurposed as an effective anti-parasitic and anti-fungal compound with high selectivity.

## MATERIALS AND METHODS

### Pathogen culture.

Culture conditions for all organisms used in this study are summarized in Table S4.

### Drug sensitivity assays.

The cytotoxicity effect of MitoTam was tested in a variety of organisms. Briefly, cells were seeded in 96-well plates at concentrations summarized in Table S4 and grown under standard conditions for 24 to 120 h, as indicated. The drug was serially diluted in a medium using a 2-fold dilution across a plate, resulting in a total volume of 200 μL per well. Each experiment included untreated control cells, as well as a positive control treated with a known growth inhibitory compound for each respective pathogen. The results were statistically analyzed, and dose-response curves plotted using Prism 6.01 (GraphPad Software) and expressed as the half-maximal effective concentration (EC_50_).

The growth of C. albicans and C. neoformans cultures was determined from OD_600_ values measured on the I-Mark microplate reader (Bio-Rad). Plasmodium falciparum dose-response curves were generated using SYBR green I, as described previously (58). In brief, triplicate 2-fold compound dilution series were set up in 100 μL parasite cultures ( ~0.2% parasitemia in 2% hematocrit). After two cycles (96 h), parasites were lysed with 20 μL of 6× SYBR green I lysis buffer (0.16% saponin; 20 mM Tris-HCl, 5 mM EDTA, 1.6% Triton X-100, pH 7.4), supplemented with 1:1000 SYBR green I (from 10000× stock, Thermo Fisher). Fluorescence intensity was measured with an Infinite M200 Pro Multimode Microplate Reader (Tecan). Trypanosoma cruzi epimastigote cell growth was assessed by CellTiter-Glo 2.0 cell viability assay in a 96-well plate according to the manufacturer’s protocol. As indicated, the dose-response curves of T. brucei and other pathogens were plotted from cell counts performed on the Guava EasyCyte 8HT flow cytometer (Luminex). The instrument setting and gate selection were adjusted using untreated control for each organism. For *G. intestinalis*, A. castellanii, and N. fowleri, that tend to adhere to the walls of the cultivation vessel, were placed on ice for 20 min and subsequently paraformaldehyde was added to a final concentration of 2% before counting the cells on the flow cytometer.

### Trypanosoma brucei growth analysis.

T. brucei bloodstream cells were seeded at a concentration of 2 × 10^5^ and 8 × 10^5^ cells/mL of 5 mL HMI-9 medium in aerobic culture flasks. Biological triplicates were analyzed, including untreated control cultures and cultures treated with final concentrations of 40 nM and 100 nM MitoTam, respectively. Cells were grown under standard growth conditions (37°C, 5% CO_2_). At each time point (8, 12, 16, 24, and 48 h), 20 μL were sampled, diluted 10× in fresh HMI-9, and the culture concentration was assessed using Guava EasyCyte 8HT flow cytometer (Luminex). The count parameters and gates were set according to previously measured cultures. In parallel, cells were checked under the microscope at each time point to confirm that live, moving cells were present in each culture.

### Trypanosoma brucei dead cell staining.

T. brucei BSF cells were seeded at a concentration of 2 × 10^5^ cells/mL in 5 mL HMI-9 medium in aerobic cultivation flasks. Biological triplicates were set up, including an untreated control group and groups treated with final concentrations of 40 nM and 100 nM MitoTam. The cells were grown under regular growth conditions. At each time point (8, 12, 16, and 24 h), 20 μL were sampled from each flask, 10× diluted in fresh HMI-9 to a final volume of 200 μL and 2 μL of SYTOX Green Dead Cell Stain (Invitrogen) was added. Cells were incubated at standard growth conditions for 30 min, after which live cell counts were analyzed using Guava EasyCyte 8HT flow cytometer (Luminex). The count parameters and gates were set according to previously measured cultures.

### Measurement of ROS production in T. brucei.

Intracellular and intramitochondrial ROS production was monitored using published protocols (59). Intracellular ROS levels were evaluated using three biological replicates of approximately 1 × 10^6^ untreated cells and cells treated with 100 nM MitoTam for 12 h. The samples were collected, incubated with 10 μM 2′,7′-dichlorofluoresceine diacetate (DCFH-DA, Sigma-Aldrich) for 30 min and washed with PBS-G (1× PBS with 6 mM glucose). Using a 488 nm excitation laser and a 530/30 nm detector, 10,000 events were immediately counted on the BD FACSCanto II instrument.

Mitochondrial ROS production was assessed using the MitoSOX indicator (Thermo Fisher Scientific) in untreated cells and cells treated with 40 nM or 100 nM MitoTam. An equal number of cells (1 × 10^6^) for each treatment was collected, washed in PBS-G, resuspended in HMI-9 medium with 5 μM MitoSOX and stained for 30 min at 37°C, 5% CO_2_. After staining, cells were spun down, resuspended in PBS, and immediately analyzed by flow cytometry (BD FACS Canto II Instrument, 488 nm excitation laser and 585/15 nm emission). For each sample, 10 000 events were collected. All values were plotted and statistically analyzed using Prism (8.0) (GraphPad Software).

### Comparative label-free proteomics.

For T. brucei bloodstream forms, a final concentration of 100 nM MitoTam was added to approximately 3 × 10^7^ cells in the exponential growth phase and cultures incubated for 14 h. Cell viability was checked microscopically before harvesting and washing three times in PBS (1200g, 10 min, 4°C). An untreated control group was prepared in parallel. Both groups were prepared in three biological replicates. The pellets were subjected to reductive alkylation and tryptic digest using routine procedures. Peptides were then analyzed by liquid chromatography-tandem mass spectrometry on an ultimate3000 nano rapid separation LC system (Dionex) coupled to an Orbitrap Fusion mass spectrometer (Thermo-Fisher Scientific). Data were analyzed as described in (45, 60) using label-free quantification in MaxQuant (43) searching the TriTrypDB T. brucei strain TREU927 protein database version 54 (61).

For C. albicans, approximately 1 × 10^4^ cells were inoculated in RPMI medium, as described in (62) and left to grow for 24 h at 35°C without agitation. MitoTam was added to the final concentration of 4.4 μM and cultures incubated for 12 h. Cells were then checked under a microscope, harvested, washed three times with PBS (1 200g, 10 min, 4°C). Pellets were then lyzed using FastPrep 24 5G homogenizer (MP Biomedicals) according to the manufacturer’s instructions. Lysates were used for the label-free proteomic analysis and compared with the untreated control group prepared in parallel. Both groups were prepared in three biological replicates. Analysis was performed based on the C. albicans protein database downloaded from Uniprot on 12.11.2019 (63). The threshold settings for comparative proteomic analyses and data processing were chosen as described in (45) for both organisms. Briefly, thresholds were set to Q-value = 0, unique peptides detected >2 and the protein had to be identified at least twice in one of the conditions. For proteins found under only one condition, the average log_2_ converted intensity of 23 was selected as a minimum threshold. Significantly changed proteins were considered only if the fold change was >2.0.

In the proteomic results of C. albicans, localization was predicted based on Uniprot (63). For T. brucei proteomic analysis, manual annotation and localization prediction were based on T. brucei 927 database or T. brucei bloodstream form mitoproteome database obtained from (46). Volcano plots were drawn in Perseus 1.6.15.0 (44). At first, proteins only identified by site, reverse, potential contaminants, and proteins detected in less than two experiments within at least one group were excluded. The imputation was performed using a normal distribution with width 0.3 and downshift of 1.8, separately for each column. The parameters of the volcano plot were set up as following: statistical *t* test for both sides, 250 randomizations, false discovery rate of 0.05 and S_0_ of 0.1.

### Mitochondrial membrane potential measurements.

The mitochondrial membrane potential (ΔΨm) of BSF T. brucei cells was estimated using the red fluorescent stain MitoTracker Red CMXRos (Invitrogen). MitoTam was added to 5 mL T. brucei culture in an exponential growth phase at a final concentration of 1 μM and cells were incubated for 1 h under standard conditions. Subsequently, MitoTracker Red CMXRos was added to a final concentration of 100 nM and samples were incubated for another 10 min. Cultures were spun (1,300 g, 10 min, RT), resuspended in 5 mL of growth medium with 1 μM MitoTam and left for an additional 20 min under standard growth conditions. An untreated control was prepared in parallel. Treated and untreated cells were washed and transferred to 300 μL PBS, spread on microscopy slides, and left to settle. The slides were fixed by immersion in ice-cold methanol for 10 min, once the excess methanol had evaporated, the slides were mounted using Vectashield with DAPI (Vector laboratories), covered with cover slides, and sealed using nail varnish. The slides were imaged on a Leica TCS SP8 WLL SMD-FLIM microscope, using LAS X 3.5.6 imaging software. All images were captured using the exact same microscope setting. Quantification and comparison of signals were performed in NIS-Elements 5.30 (Nikon). Briefly,10 to 20 z-stacks were captured using LAS X automatic settings. A single stack with the highest overall intensity was chosen from the batch, and using the Artificial Intelligence module, areas of signal corresponding to individual cells were mapped and the average intensity was determined from the selected area. Furthermore, cell DNA visualized by DAPI was also quantified in the same manner and compared across all images as a reference value. The signal intensity of MitoTracker Red CMXRos was then compared in treated and untreated control, while the intensity of the DAPI signal was used to confirm the reproducibility of the reading.

Estimation of ΔΨm in live cells was performed as described previously (59). Briefly, an equal number of cells (1 × 10^6^) for each treatment was spun (1,400 g, 10 min, RT), the pellets were resuspended in HMI-9 medium with 60 nM TMRE (Thermo Fisher Scientific T669) and stained for 30 min at 37°C, 5% CO_2_. After staining, cells were spun down (1,400 g, 10 min, RT) and resuspended in 2 mL of PBS and immediately analyzed by flow cytometry (BD FACS Canto II Instrument). For each sample, 10,000 events were collected. As a control for mitochondrial membrane depolarization, cells were treated with 20 μM protonophore FCCP (carbonyl cyanide 4-[trifluoromethoxy] phenylhydrazone, Sigma). Data were evaluated using BD FACS Diva software (BD Company) and further analyzed using Prism (8.0) (GraphPad Software).

Estimation of the ΔΨm was also performed using the fluorescent probe Safranin O (Sigma) according to (59). From each treatment, 2 × 10^7^ cells were collected, spun down (1,400 g, 10 min, RT), and resuspended in ANT buffer (8 mM KCl, 110 mM potassium gluconate, 10 mM NaCl, 10 mM HEPES, 10 mM K_2_HPO_4_, 0.015 mM EGTA, 0.5 mg/mL fatty acid-free BSA, 10 mM mannitol, 1.5 mM MgCl_2_) with 5 μM Safranin O. Intact live cells were permeabilized by the addition of 4 μM digitonin (Calbiochem). The fluorescence of all samples was measured at RT and constant stirring and recorded on a Hitachi F-7100 spectrofluorometer (Hitachi High Technologies) at a 5 Hz acquisition rate, using 495 and 585 nm excitation and emission wavelengths, respectively. As indicated, the reaction was started by adding 1 mM ATP (PanReac AppliChem), F_o_F_1_-ATP synthase substrate, and stopped by the addition of 10 μg/mL oligomycin (Sigma), F_o_F_1_-ATP synthase inhibitor. The protonophore SF6847 (Enzo Life Sciences) was added at a final concentration of 250 nM as control for maximal depolarization. Fluorescence data were analyzed using Prism (8.0) (GraphPad Software).

### High-resolution respirometry.

The effect of MitoTam on respiration was analyzed by Oroboros Oxygraph-2K (Oroboros Instruments Corp., Innsbruck, Austria) as described (59). Bloodstream form T. brucei cells were incubated with 40 nM or 100 nM MitoTam for 16 h and 24 h, as indicated. For each treated sample and control, 2 × 10^7^ cells were spun down (1,400 g, 10 min, RT) and pellets were washed in Mir05 medium (0.5 mM EGTA, 3 mM MgCl_2_, 60 mM lactobionic acid, 20 mM taurine, 10 mM KH_2_PO_4_, 20 mM HEPES, 110 mM sucrose, 1 mg/mL fatty acid-free BSA, pH 7.1). Before the measurement started, the pellets were resuspended in 0.5 mL of Mir05 medium preheated to 37°C and transferred to the respiration chamber. Respiration was monitored at 37°C and with constant stirring. The experiment started with the addition of 10 mM glycerol-3-phosphate (Sigma), the mitochondrial glycerol-3-phosphate dehydrogenase substrate, and respiration was inhibited by the addition of 250 μM SHAM (Salicylhydroxamic acid), the inhibitor of the trypanosomal alternative oxidase. The acquired data were analyzed using Prism (8.0) (GraphPad Software).

### Western blot analysis.

T. brucei BSF cells were incubated with 100 nM MitoTam and harvested at different time points after addition as indicated (0h, 8h, 12h, 16h and 24h). Cells were spun down (1,400 g, 10 min, RT), pellets were washed in PBS (pH 7.4) and resuspended in 3 × SDS-Page sample buffer (150 mM Tris pH 6.8, 300 mM 1,4-dithiothreitol, 6% (wt/vol) SDS, 30% (wt/vol) glycerol, 0.02% (wt/vol) bromophenol blue). The whole-cell lysates were denatured at 97°C for 8 min and stored at −80°C. For Western blot analysis, a volume of sample equal to 2.5 × 10^6^ cells per well was loaded onto 12% gel, separated by SDS-Page, blotted onto a nitrocellulose membrane (Amersham Protram 0,2 μm PC GE Healthcare Life Sciences) and probed with a monoclonal (MAb) or polyclonal antibody (pAb). Incubation with primary antibodies was followed by a secondary antibody, either HRP‐conjugated goat anti‐rabbit or an anti‐mouse antibody (1:5,000, Bio-Rad). Antibodies were detected using the enhanced chemiluminescence system (Immobilon Forte Western HRP substrate, Merck) on the Amersham Imager 600 (GE Health Care Life Sciences). The PageRuler™ Plus prestained protein ladder (Thermo Fisher Scientific 26619) was used to determine the size of the detected bands. Primary antibodies were: MAb anti-Hsp70 (1: 2,000), MAb anti-AOX (1:1,000), pAb anti-AAC (1:1,000), anti-VDAC (1:1,000), pAb anti-PYK (pyruvate kinase) (1:1,000), pAb anti-APRT (adenine phosphoribosyl transferase) (1:1,000) and antibodies against F_o_F_1_-ATP synthase subunits β (1:2,000), p18 (1:1,000), Tb1 (1:1,000), Tb2 (1:1,000), and OSCP (1:1,000) (59, 64–66).

### Measurement of the ATP/ADP ratio and total cellular ATP levels.

Changes in the ATP/ADP ratio and total cellular ATP were determined in BSF T. brucei cells using the d-luciferin-luciferase bioluminescent enzymatic reaction (assay kit Sigma MAK135) according to manufacturer’s instructions. Briefly, from each sample 1 × 10^6^ cells were harvested and washed once with PBS supplemented with 6 mM glucose (PBS-G). Pellets were resuspended in PBS-G and the mixture transferred into a microtiter plate (96-well white flat-bottom). The bioluminescence signal was recorded in an Orion II microplate luminometer (Titertek Berthold) and analyzed using Prism (8.0) (GraphPad Software).

### *In situ* measurement of ATP levels.

Cytosolic and mitochondrial ATP was measured using BSF T. brucei cell lines expressing firefly luciferase with or without N-terminal mitochondrial localization signal (MLS) following published protocols (67). Briefly, MitoTam treated cells, as well as untreated control, were spun (1 × 10^7^ cells) (1,400 g, 10 min, RT) and washed in PBS-G. The pellets were resuspended in HEPES-LUC+Glu buffer (10 mM d-glucose, 20 mM HEPES, 116 mM NaCl, 5.6 mM KCl, 8 mM MgSO_4_, 1.8 mM CaCl_2,_ pH 7.4) and transferred to a 96-well microtiter plate. ATP-dependent luciferase bioluminescence was recorded on a plate reader (Tecan Infinite M100). The light emission was started with the addition of a d-luciferin solution (50 μM; Sigma), collected for 5 min, and statistically analyzed using Prism (8.0) (GraphPad Software).

### Determination of the mitochondrial membrane integrity.

T. brucei bloodstream mitochondria were isolated by digitonin fractionation, according to (68). Briefly, approximately 1 × 10^8^ cells were harvested (1400 g, 10 min, RT) and transferred to Hanks’ balanced salt solution (Sigma-Aldrich). The protein concentrations of the samples were determined by the BCA assay kit (Sigma-Aldrich, USA). Digitonin (Calbiochem) was added to a protein:mass ratio of 1:0.15, lysate was incubated for 4 min and then spun. The pellet was washed and used as a mitochondria-enriched fraction, while the supernatant was used as a cytosolic fraction. Activities of two marker enzymes were used to assess the purity of fractions and intactness of mitochondrial membranes. The enzyme activities of cytosolic pyruvate kinase (PYK) and mitochondrial threonine dehydrogenase (TDH) were assayed spectrophotometrically at 340 nM by monitoring NADH concentration changes during the reaction. The activity of PYK was measured according to (69) in 0.1 M TEA buffer (ThermoFisher Scientific) (final pH 7.6), 5 mM MgSO_4_ and 50 mM KCl, with the addition of 2.8 mM phosphoenolpyruvate, 2 mM ADP, 0.3 mM NADH and lactate dehydrogenase. The activity of TDH was measured in 0.2 M Tris-HCl buffer with 0.25 M KCl (final pH 8.6), with the addition of 120 mM threonine and 2.5 mM NAD^+^. Different concentrations of MitoTam were added to the reaction and TDH activity was monitored as a marker for disruption of the mitochondrial membranes. Treatment with nonionic detergent Triton X-100 was used to completely disrupt mitochondrial membranes and thus release maximum TDH activity. Data were statistically analyzed and plotted with Prism (8.0) (GraphPad Software).

### Membrane fluidity expressed as the regiospecific anisotropy of fluorescence polarization.

Artificial membranes were prepared via hydration of lipide film (2.5 mg, 0.0034 mmol DPPC + MitoTam 10 mol.% + 0.1 mol% DPH or 0.2 mol % TMA-DPH) with physiological saline (2 mL). The lipide solution was extruded through polycarbonate membrane with pore size 0.4 μm. An 100 μL aliquot of liposomal solution was diluted with additional 900 μL of physiological saline to give clear solution of subvisible particles (≈ 0.125 mg/mL). Fluorescence anisotropy was measured over range of temperatures (22 – 49°C) in 3°C increments while the slides in the spectrometer FLS1000 were set to 1 to 3 nm. In between the individual temperature measurements was the sample stirred with magnetic stir bar. Each measurement was average of three subsequent scans and the whole data set was collected as triplicate (DPH excitation 360 nm, emission 435 nm; TMA-DPH excitation 365 nm, emission 425 nm). Fluorescence anisotropy (r) is calculated automatically by software provided with the instrument, according to r = (Ivv−IvhG)/(Ivv + 2IvhG), where Ivv and Ivh are the intensities of the vertically and horizontally polarized components of the fluorescent light, respectively, after excitation with vertically polarized light. G = Ihv/Ihh is a grating correction factor for the optical system.

### Survival analysis in a mouse model.

To evaluate the trypanosomiasis effect of MitoTam on the mortality in mice, a group of 14 BALB/c mice was infected IP with approximately 2 × 10^5^ BSF T. brucei cells strain STIB920 and the group of 14 mice was infected with 5 × 10^5^ BSF cells of T. brucei *brucei* 427 strain, both in 100 μL sterile PBS. Mice were monitored every 12 h. Based on previous experience with the progression of the disease, half of the infected mice from each group were IV injected with MitoTam (3 mg/kg bw) on days 3 and 5 after infection. The survival of the mice was visually monitored for up to 13 days after infection, the day of death was recorded for each animal.

### T. brucei mouse infection model.

To assess the effect of MitoTam on the morbidity of trypanosomiasis in mice, 16 BALB/c mice were infected with 2 × 10^5^ BSF T. brucei cells (strain STIB920) in 100 μL of sterile PBS. On day 2 and 4 postinfection, eight mice were injected with MitoTam at a final concentration of (3 mg/kg bw).

Mice were monitored daily, and blood samples were collected from a tail prick from each animal. Blood drops were smeared on microscopy slides and stained using Wright-Giemsa stain modification, Diff-Quick (Medion Diagnostics) according to the manufacturer’s protocol. From each slide three images of random places, where red blood cells were not overlaying, were taken using an inverted widefield microscope Eclipse Ti2 (Nikon) using the CFI Plan Apochromat Lambda 20× objective (Nikon) with NIS-Elements AR 5.20 (Nikon). The images were then processed using the Artificial Intelligence module on NIS-Elements 5.30 (Nikon), manually trained to detect and quantify the number of red blood cells and trypanosomes. The ratio of trypanosomes to red blood cells was calculated and used to plot the data and calculate the statistical difference (Student's *t* test) between the treated and untreated groups in a given days. Data were plotted with Prism (8.0) (GraphPad Software). Animal handling was approved by the Czech Ministry of Agriculture (53659/2019-MZE-18134).

### L. mexicana mouse subcutaneous leishmaniasis model.

The culture conditions of the L. mexicana promastigotes were as indicated in Table S4, their concentration was determined by hemocytometer. Before infection experiments, promastigotes were harvested, washed three times and resuspended in PBS. Twenty BALB/c mice (10 weeks old) were anesthetized IP with a mixture of ketamine (150 mg/kg) and xylazine (15 mg/kg) and infected intradermally in the ear pinnae by injection of 10^7^ promastigotes. For a group of 10 animals, MitoTam was administered IP at 1, 2, 3, 5, 6 and 7 weeks after infection at a dose of (3 mg/kg body weight), other 10 animals served as nontreated controls. The presence of lesions was monitored for 12 consecutive weeks.

### Rescue assay for *Leishmania* spp. amastigotes.

Initially, murine macrophage cells (J774) were seeded in RPMI supplemented with 10% FCS and 50 μg/mL phorbol 12-myristate 13-acetate and left to differentiate for 24 h. Then, after washing the cells once with warm (37°C) serum-free RPMI, stationary phage promastigotes of L. infantum or L. major were added in a 10:1 ratio (2.5 × 10^6^ cell/mL). After 24 h of incubation, cells were washed five times with serum-free RPMI and exposed to increasing concentration of MitoTam (or amphotericin B, as positive control) in RPMI (2% FCS). After 2 days, macrophages were l ysed with 20 μL of 0.05% SDS in RPMI for 30 s and released *Leishmania* cells were incubated with M199 10% FCS for another 3 days. Finally, the viability of transformed live cells was determined by the resazurin viability assay.
