# Supplementary material for: Repurposing of MitoTam: Novel Anti-Cancer Drug Candidate Exhibits Potent Activity against Major Protozoan and Fungal Pathogens
Source: Antimicrob Agents Chemother. 2022 Jul 20;66(8):e00727-22. doi: 10.1128/aac.00727-22 (PMC9380531; doi:10.1128/aac.00727-22)
Supplement: Supplemental file 1 — Table S1, Fig. S2 to S4, descriptions of Tables S2 to S4. Download aac.00727-22-s0001.pdf, PDF file, 1.69 MB [file aac.00727-22-s0001.pdf]

Supplementary data:

Table S1: Table of screened pathogens summarizing their impact on global health.

Table S2: ***C. albicans*** **proteomic analysis after 12 h of MitoTam exposure.**

Proteome comparison of *C. albicans* cultivated in the presence of 4.4 µM MitoTam for 12 h and untreated culture. The table is organized in four sheets: All detected proteins, Downregulated in MitoTam, Upregulated in MitoTam and Raw data. The first three sheets are showing fold-abundance change only for clarity. Upregulated and downregulated proteins were filtered by >2-fold change. Proteins were identified and, where applicable, subcellular localization was annotated based on Uniprot [63].

Table S3: ***T. brucei*** **proteomic analysis after 14h of MitoTam exposure.**

Proteome comparison of *T. brucei* cultivated in the presence of 100 nM MitoTam for 14 h and untreated culture. Table is organized in four sheets: All detected proteins, Downregulated in MitoTam, Upregulated in MitoTam and Raw data. The first three sheets are simplified to demonstrate fold-abundance change only for clarity. Upregulated and downregulated proteins were filtered by >2-fold change. Where applicable, manual annotation and localization prediction was based on the *T. brucei* 927 mitochondrial proteome [46].

Table S4: Summary of culture conditions and conditions for viability assays for all organisms used in this study.

Figure S1: Dose-response curves for MitoTam, Tamoxifen and control compounds.

Cells were incubated with increasing concentrations of MitoTam, tamoxifen or control compounds under cultivation conditions described in Table S4. Cell concentration, viability or absolute OD_{595} values were plotted against a compound concentration, dose-response curves and mean IC50 values (summarized in Table 1) were generated in Prism (8.0) (GraphPad Software).

Figure S2: *Leishmania* sensitivity towards MitoTam evaluated by the intramacrophage assay.

Murine macrophage cell culture J774A.1 was infected with amastigotes of *L. major* (pink line) or *L. infantum* (brown line) and incubated with increasing concentration of MitoTam as indicated on the x-axis. Uninfected macrophages (green line) were included as a control.

Figure S3: Survival analysis of BALB/c mice infected with *T. brucei.*

Survival of infected mice was monitored daily for 8 days. As depicted (dashed lines) half of the infected mice were injected with MitoTam (+MitoTam) on days 2 and 4 post-infection (3 mg/kg body weight). Number of surviving mice from MitoTam treated group (red line) as well as from the untreated control group (-MitoTam) (blue line) are depicted.

Figure S4: MitoTam alters the mitochondrial function of bloodstream *T. brucei*.

Cells were incubated with 40 nM of MitoTam for 16 and 24 hours and their mitochondrial parameters were assayed as for 100 nM MitoTam.

A) The O_2 flux per cell using high-resolution respirometry after addition of glycerol-3-phosphate was determined in BSF *T. brucei* untreated control cells (-MT, blue) and BSF cells treated with 40 nM

MitoTam for 16 hours (+MT 16 h, red) and 24 hours (+MT 24 h, red) (box and whiskers plot, *n* = 3, **p < 0.01, ***p < 0.001).

B) Relative ADP/ATP ratio analyzed using a bioluminescence assay kit in BSF *T. brucei* untreated control cells (-MT, blue) and BSF cells treated with 40 nM MitoTam for 16 hours (+MT 16 h, red) and 24 hours (+MT 24 h, red) (box and whiskers plot, *n* = 3).

C) Cytosolic and mitochondrial ATP levels were assessed in transgenic BSF *T. brucei* cell lines expressing firefly luciferase. Results of untreated control cells (-MT, blue) were compared with results of cultures treated with 40 nM MitoTam for 16 hours (+MT 16 h, red) and 24 hours (+MT 24 h, red). Data were normalized to the respective values of the untreated control cells and expressed as a percentage (mean ± s.d., *n* = 4).

D) Total cellular ATP levels in BSF *T. brucei* were determined using a bioluminescence assay kit. Data from cultures treated with MitoTam for 16 hours (+MT 16 h, red) and 24 hours (+MT 24 h, red) were normalized to the values of the untreated control cells (-MT, blue) and expressed in percentage (box and whiskers plot, n = 4).

E) Flow cytometry of TMRE stained cells was used to determine $\Delta\Psi_m$ of BSF *T. brucei*. Data from cultures treated with 40 nM MitoTam for 16 hours (16 h +MT, red) and 24 hours (+MT 24 h, red) were normalized to the values of the untreated control cells (-MT, blue) and expressed in percentage. Uncoupler FCCP was added as a control for $\Delta\Psi_m$ depolarization (box and whiskers plot, n = 6, ****P <0.0001).

F) *In situ* $\Delta\Psi_m$ was measured in digitonin-permeabilized BSF *T. brucei* cells stained with Safranin O dye. Where indicated, the F_oF_1-ATP synthase substrate – ATP, the F_oF_1-ATP synthase inhibitor - oligomycin (Olgm) and the protonophore SF6847 were added. Representative traces from measurement of untreated control cells (-MT, blue) in comparison with cells treated with 40 nM MitoTam for 16 hours (+MT 16 h, orange) and 24 hours (+MT 24 h, red) are shown.

Tab. S1

Pathogen	Ilnesses	Estimated Annual Incidence/human infections reported	Mortality rate	Treatment options	References
Trypanosoma brucei	Human African trypanosomiasis (sleeping sickness)	≤1000	~100%	fexinidazole, pentamidine, nifurtimox–eflornithine combination therapy, suramin, melarsoprol	[1]
Trypanosoma cruzi	Chagas disease	30 000	1-50%	benznidazole, nifurtimox	[2][3][4]
Leishmania major, Leishmania mexicana	cutaneous leishmaniosis	600 000 -1 000 000	N/A	for immunocompetent patients -no treatment or topical oitments / for immunocompromised patients - sodium stibogluconate, miltefosine, fluconazole	[5][6]
Plasmodium falciparum	malaria	214 000 000	10-50%	artemisinin-based combination therapy	[7]
Candida albicans	superficial mucosal and dermal infections, disseminated systemic invasive candidiasis	750 000 (invasive candidiasis)	30 - 70%	antibiotics (echinocandin, fluconazole, or amphotericin B)	[8][9][10]
Cryptococcus neoformans	cryptococcosis	223 000	24-95%	anti-fungal drugs (amphotericine B, fluconazole, itraconazole)	[11][12]
Naegleria fowleri	Primary amoebic meningoencephalitis	431 cases reported until 2020	≥ 90%	combination of antibiotics (such as amphotericin B, fluconazole, miltefosine) and drugs for relieving intracranial pressure	[13][14]
Acanthamoeba spp.	Granulomatous amoebic encephalitis (GAE) Acanthamoeba keratitis (AK)	AK around 3,000 cases reported, GAE approximately 200 cases have been described until 2004	≥ 90% (GAE)	antimicrobial drugs used alone or in combined therapy (amphotericin B, pentamidine, sulfadiazine, flucytosine, fluconazole or itraconazole, chlorhexidine gluconate, ketoconazole)/antimicrobial and anti-inflammatory medicines have to be combined with cystidal drugs	[15][16][17]
Giardia intestinalis	giardiasis	28 000 000	N/A	Metronidazole, Tinidazole, Albendazole	[18]
Trichomonas vaginalis	trichomoniasis	156 000 000	N/A	Metronidazole, Tinidazole	[19]

References:

[1] WHO, “Human African Trypanosomiasis.” [Online]. Available: https://www.who.int/health-topics/human-african-trypanosomiasis.

[2] PAHO, “Chagas in America,” 2017. [Online]. Available: https://www3.paho.org/hq/index.php?option=com_content&view=article&id=13566:chagas-in-americas&Itemid=40721&lang=en.

[3] D.-A. Álvarez-Hernández, G.-A. Franyuti-Kelly, R. Díaz-López-Silva, A.-M. González-Chávez, D. González-Hermosillo-Cornejo, and R. Vázquez-López, “Chagas disease: Current perspectives on a forgotten disease,” *Rev. Médica del Hosp. Gen. México*, vol. 81, no. 3, pp. 154–164, Jul. 2018.

[4] Z. M. Cucunubá, O. Okuwoga, M. Basáñez, and P. Nouvellet, “Increased mortality attributed to Chagas disease : a systematic review and meta-analysis,” *Parasit. Vectors*, 2016.

[5] WHO, “Leishmaniasis.” [Online]. Available: https://www.who.int/health-topics/leishmaniasis#tab=tab_3.

[6] S. Burza, S. L. Croft, and M. Boelaert, “Leishmaniasis,” *Lancet*, vol. 6736, pp. 1–20, 2018.

[7] WHO, “Malaria,” 2021. [Online]. Available: https://www.who.int/news-room/fact-sheets/detail/malaria%0D%0A.

[8] F. Bongomin, S. Gago, R. O. Oladele, and D. W. Denning, “Global and multi-national prevalence of fungal diseases—estimate precision,” *Journal of Fungi*, vol. 3, no. 4. 2017.

[9] F. Lamoth, S. R. Lockhart, E. L. Berkow, and T. Calandra, “Changes in the epidemiological landscape of invasive candidiasis,” *J. Antimicrob. Chemother.*, vol. 73, pp. i4–i13, 2018.

[10] P. G. Pappas *et al.*, “Executive Summary: Clinical Practice Guideline for the Management of Candidiasis: 2016 Update by the Infectious Diseases Society of America,” *Clinical Infectious Diseases*, vol. 62, no. 4. pp. 409–417, 2015.

[11] E. K. Maziarz and J. R. Perfect, “Cryptococcosis,” *Infectious Disease Clinics of North America*, vol. 30, no. 1. pp. 179–206, 2016.

[12] WHO, “WHO | Cryptococcal disease: what’s new and important,” *WHO*, 2018.

[13] E. Grace, S. Asbill, and K. Virga, “Naegleria fowleri: Pathogenesis, diagnosis, and treatment options,” *Antimicrobial Agents and Chemotherapy*, vol. 59, no. 11. pp. 6677–6681, 2015.

[14] S. K. Maciver, J. E. Piñero, and J. Lorenzo-Morales, “Is Naegleria fowleri an Emerging Parasite?,” *Trends in Parasitology*, vol. 36, no. 1. Elsevier Ltd, pp. 19–28, 01-Jan-2020.

[15] K. Kot, N. A. Łanocha-Arendarczyk, and D. I. Kosik-Bogacka, “Amoebas from the genus Acanthamoeba and their pathogenic properties,” *Ann. Parasitol.*, vol. 64, no. 4, pp. 299–308, 2018.

[16] G. S. Visvesvara, H. Moura, and F. L. Schuster, “Pathogenic and opportunistic free-living amoebae: Acanthamoeba spp., Balamuthia mandrillaris, Naegleria fowleri, and Sappinia diploidea,” *FEMS Immunology and Medical Microbiology*, vol. 50, no. 1. pp. 1–26, 2007.

[17] F. L. Schuster and G. S. Visvesvara, “Free-living amoebae as opportunistic and non-opportunistic pathogens of humans and animals,” *International Journal for Parasitology*, vol. 34, no. 9. Pergamon, pp. 1001–1027, 01-Aug-2004.

[18] U. Ryan, N. Hijjawi, Y. Feng, and L. Xiao, “Giardia: an under-reported foodborne parasite,” *Int. J. Parasitol.*, vol. 49, no. 1, pp. 1–11, 2019.

[19] O. T. Van Gerwen and C. A. Muzny, “Recent advances in the understanding and management of of Trichomonas vaginalis infection,” *F1000Research*, vol. 8, pp. 1–10, 2019.

Fig. S1

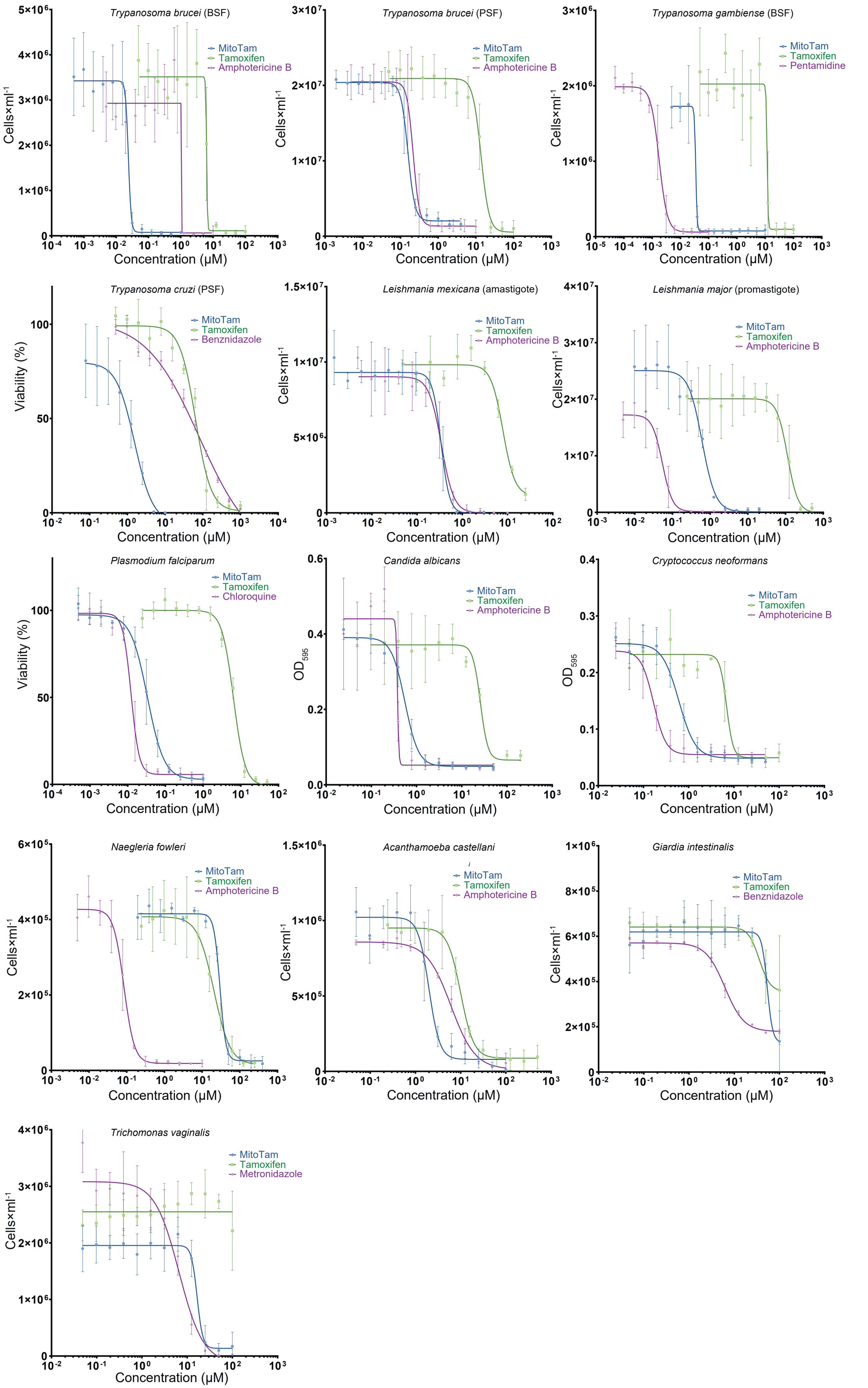
Trypanosoma brucei (BSF)
MitoTam
Tamoxifen
Amphotericine B
Cells×ml-1
Concentration (µM)
Trypanosoma brucei (PSF)
MitoTam
Tamoxifen
Amphotericine B
Cells×ml-1
Concentration (µM)
Trypanosoma gambiense (BSF)
MitoTam
Tamoxifen
Pentamidine
Cells×ml-1
Concentration (µM)
Trypanosoma cruzi (PSF)
MitoTam
Tamoxifen
Benznidazole
Viability (%)
Concentration (µM)
Leishmania mexicana (amastigote)
MitoTam
Tamoxifen
Amphotericine B
Cells×ml-1
Concentration (µM)
Leishmania major (promastigote)
MitoTam
Tamoxifen
Amphotericine B
Cells×ml-1
Concentration (µM)
Plasmodium falciparum
MitoTam
Tamoxifen
Chloroquine
Viability (%)
Concentration (µM)
Candida albicans
MitoTam
Tamoxifen
Amphotericine B
OD595
Concentration (µM)
Cryptococcus neoformans
MitoTam
Tamoxifen
Amphotericine B
OD595
Concentration (µM)
Naegleria fowleri
MitoTam
Tamoxifen
Amphotericine B
Cells×ml-1
Concentration (µM)
Acanthamoeba castellani
MitoTam
Tamoxifen
Amphotericine B
Cells×ml-1
Concentration (µM)
Giardia intestinalis
MitoTam
Tamoxifen
Benznidazole
Cells×ml-1
Concentration (µM)
Trichomonas vaginalis
MitoTam
Tamoxifen
Metronidazole
Cells×ml-1
Concentration (µM)

Fig. S2

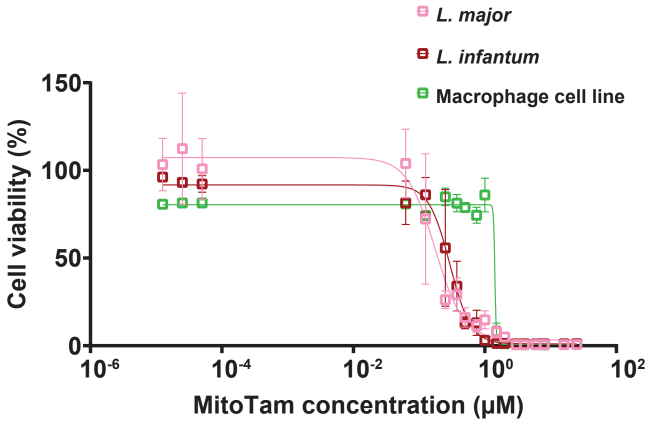
L. major
L. infantum
Macrophage cell line
Cell viability (%)
150
100
50
0
10-6
10-4
10-2
100
102
MitoTam concentration (μM)

Fig. S3

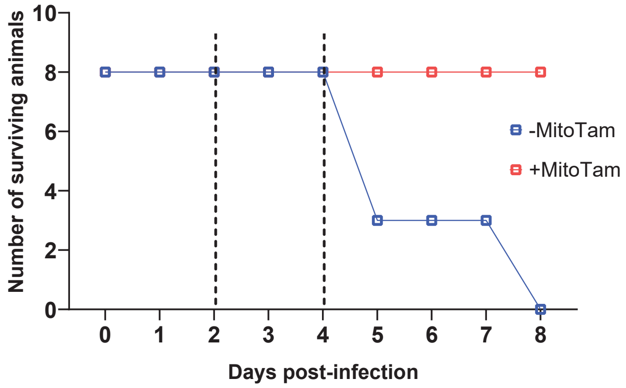

Number of surviving animals
10
8
6
4
2
0
0
1
2
3
4
5
6
7
8
Days post-infection
-MitoTam
+MitoTam

Fig. S4

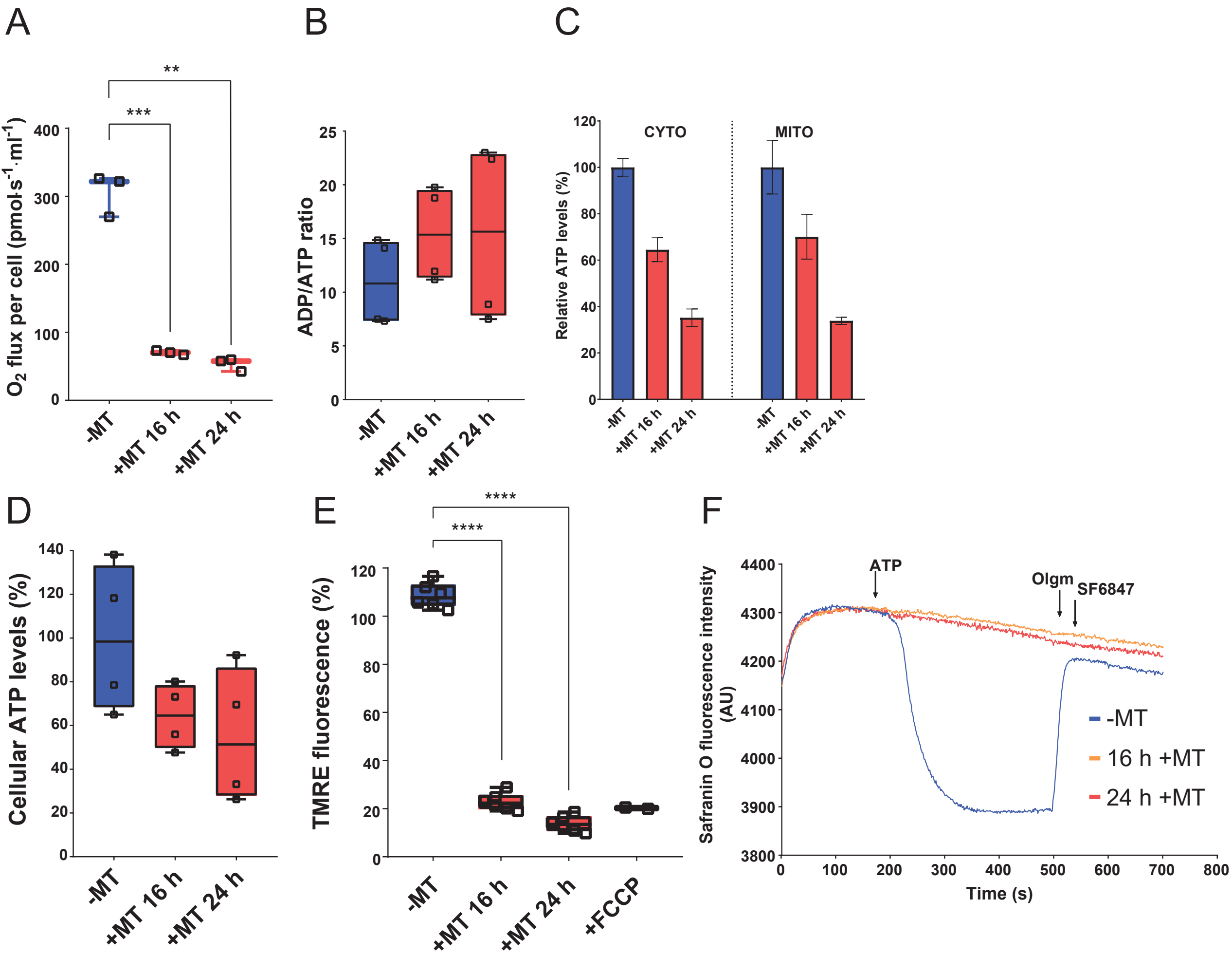
A
B
C
D
E
F
O2 flux per cell (pmol·s-1·ml-1)
ADP/ATP ratio
Relative ATP levels (%)
CYTO
MITO
Cellular ATP levels (%)
TMRE fluorescence (%)
Safranin O fluorescence intensity (AU)
Time (s)
ATP
Olgm
SF6847
-MT
+MT 16 h
+MT 24 h
+FCCP
16 h +MT
24 h +MT
***
**
****
